# Developments to improve outcomes in thyroid surgery

**DOI:** 10.1515/iss-2022-2002

**Published:** 2022-12-08

**Authors:** Thomas J. Musholt

**Affiliations:** Dept. of General, Visceral and Transplantation Surgery, University Medicine of the Johannes Gutenberg University Mainz, Mainz, Germany

Thyroid interventions are predominantly elective procedures with fortunately low mortality, but the associated surgical complications may have a significant impact on the quality of life (QOL) of patients. In Germany, 25,718 thyroidectomies and 21,320 hemithyroidectomies were performed in 2020 [1], corresponding to 72,756 recurrent laryngeal nerves (RLNs) “at risk”. Assuming a complication rate of about 0.5–5 % for permanent paresis, significant 360–3,600 new cases of RLN nerve damage occur per year in Germany alone. The complication of postoperative hypoparathyroidism after total thyroidectomy may even more severely impact the patients’ health and QOL. In the absence of a generally accepted definition, the frequency of postoperative hypoparathyroidism has so far been determined only inaccurately, based on surrogate parameters such as recorded postoperatively continued calcium medication. However, it is well known that thyroid surgery is a major cause of permanent hypoparathyroidism (>75% of cases). If one estimates a complication rate of about 1–10% after thyroid surgery, in Germany alone, 260–2,570 patients newly suffer from hypoparathyroidism each year. Staggering numbers.

This issue of Innovative Surgical Science summarizes current developments in the analysis and prevention of complications in thyroid surgery. To avoid especially bilateral RLN paresis, intraoperative neuromonitoring in intermittent form (iIONM) is widely used by thyroid surgeons. Continuous neuromonitoring (cIONM), on the other hand, is not yet as wide-spread or as consistently used in every single thyroid procedure. A major reason for the hesitance of surgeons to use cIONM is the necessary partial circular exposure of the vagus nerve in its cervical course, which potentially represents an additional operative risk. In this issue, Sinclair et al. describe a new method of intraoperative neuromonitoring that exploits the laryngeal adductor reflex and thus eliminates the need for direct stimulation of the vagus nerve. This innovation has the potential to simplify continuous neuromonitoring, to introduce cIONM in minimally invasive procedures, such as transoral thyroid surgery via a vestibular approach – described by Karakas et al. in this issue – or to enable cIONM in other surgical procedures, such as esophageal resections.

In their review article, Demarchi et al. describe results of autofluorescence imaging of parathyroid glands, including intraoperative angiography with indocyanine green. The rate of postoperative hypoparathyroidism can be effectively reduced with this new technique. Currently, the method is only used in a few clinics due to the high acquisition costs of the device. However, the technique may become established in the long-term, in a comparable way that intraoperative neuromonitoring has conquered the operating room.

Measures to improve the quality of surgical results begin with the correct (individualized) indication for surgery. With a proportion of 20–40% of patients, thyroid surgery is performed due to a suspicion of malignancy that cannot be ruled out otherwise. The high number of these so-called “diagnostic operations” has been criticized in recent years. However, the extent to which these thyroid procedures are indeed avoidable is not transparent, since especially in goiter-endemic areas there are often several reasons for surgery. Detailed information about the indications for surgery are not recorded in secondary data such as the usual medical databases (e.g., DRG statistics) or medical registers. Dedicated surgical registries were therefore established to analyze the quality of care, to identify starting points for improvement, and to carry out studies to evaluate new methods. For endocrine surgery, the Eurocrine^®^ registry, which is available throughout Europe, was established. Based on the suspicion of malignancy for a thyroid node, preoperative diagnostic procedures such as sonography and fine needle aspiration biopsy (FNAB) can be recorded in this registry.

Up to now, evaluations of diagnostic procedures before thyroid surgery were predominantly unicentric results, were limited to endocrine centers, or were carried out within the somewhat artificial environment of controlled studies. As a result of these assessments, false negative results of FNABs in Bethesda category II (benign findings) were reported to be less than 5% (that is, a malignancy was overlooked) [2]. In this issue, Staubitz et al. reveal, using “real world” data of about 8,800 patients from the Eurocrine^®^ registry, that the accuracy of FNAB in clinical routine is apparently lower than expected. In Bethesda category II, an unexpected malignancy was diagnosed postoperatively in astonishing 20.2% of cases. After excluding papillary microcarcinoma, it was still as high as 13.3%. The manifold reasons for these findings are discussed and must be differentiated in further studies which will include even more data. In addition, it becomes clear that despite a conclusive cytology result, an adequate operation is not always performed, leading to theoretically avoidable secondary interventions. In other words, currently, surgical indication mainly based on FNAB cytology remains a double-edged sword.

Besides more accurate node selection for puncture and improved training of cytologists as corrective measures, in the future FNAB results will profit from (more widespread) molecular genetic analysis of the aspirate. The new classification of thyroid neoplasms by the WHO, expected in 2024, will require molecular genetic information on the thyroid tumor (as well as a recording of the Ki-67 index and morphological aspects such as the presence of a node capsule and a possible angioinvasion) [3]. If this hopefully increases the extent of molecular analysis already been carried out preoperatively from FNABs, surgeons will be given additional preoperative diagnostic results and therefore the opportunity to an even more differentiated surgical approach. In a future optimized treatment strategy, patients with BRAF-like tumors will undergo more aggressive treatment than patients with RAS-like tumor expression patterns.
